# Plasma Gelsolin Depletion and Circulating Actin in Sepsis—A Pilot Study

**DOI:** 10.1371/journal.pone.0003712

**Published:** 2008-11-12

**Authors:** Po-Shun Lee, Sanjay R. Patel, David C. Christiani, Ednan Bajwa, Thomas P. Stossel, Aaron B. Waxman

**Affiliations:** 1 Pulmonary and Critical Care Division, Brigham and Women's Hospital, Boston, Massachusetts, United States of America; 2 Harvard Medical School, Boston, Massachusetts, United States of America; 3 Translational Medicine Division, Brigham and Women's Hospital, Boston, Massachusetts, United States of America; 4 Division of Pulmonary and Critical Care Medicine, University Hospitals of Cleveland, Case Western Reserve University, Cleveland, Ohio, United States of America; 5 Pulmonary and Critical Care Unit, Massachusetts General Hospital, Boston, Massachusetts, United States of America; Oregon Health & Science University, United States of America

## Abstract

**Background:**

Depletion of the circulating actin-binding protein, plasma gelsolin (pGSN) has been described in septic patients and animals. We hypothesized that the extent of pGSN reduction correlates with outcomes of septic patients and that circulating actin is a manifestation of sepsis.

**Methodology/Principal Findings:**

We assayed pGSN in plasma samples from non-surgical septic patients identified from a pre-existing database which prospectively enrolled patients admitted to adult intensive care units at an academic hospital. We identified 21 non-surgical septic patients for the study. Actinemia was detected in 17 of the 21 patients, suggesting actin released into circulation from injured tissues is a manifestation of sepsis. Furthermore, we documented the depletion of pGSN in human clinical sepsis, and that the survivors had significantly higher pGSN levels than the non-survivors (163±47 mg/L vs. 89±48 mg/L, p = 0.01). pGSN levels were more strongly predictive of 28-day mortality than APACHE III scores. For every quartile reduction in pGSN, the odds of death increased 3.4-fold.

**Conclusion:**

We conclude that circulating actin and pGSN deficiency are associated with early sepsis. The degree of pGSN deficiency correlates with sepsis mortality. Reversing pGSN deficiency may be an effective treatment for sepsis.

## Introduction

Plasma gelsolin (pGSN) is the secreted isoform of cytoplasmic gelsolin (cGSN), an intracellular actin-binding protein that regulates cell motility [1]. pGSN circulates in normal plasma at 190–300 mg/L [2]. Similar to cGSN, pGSN also binds actin, a major body protein that may be exposed or released by cellular injury. The consistent observation of lowered levels of pGSN in diverse states of acute injury and inflammation, such as hepatic failure, malaria, acute lung injury, myonecrosis, and cardiac injury [3]–[6], has led to a hypothesis that it participates in the clearance of actin from the circulation [2]. Further studies have revealed that critical extents of pGSN depletion in patients subjected to trauma, burns, major surgery or hematopoietic stem cell transplantation correlate with poor outcomes, including death [7]–[9]. In addition, the finding that pGSN binds inflammatory mediators such as platelet activating factor and lysophosphatidic acid suggests that its physiological function may be to localize inflammation and blunt its systemic effects, and that extensive pGSN depletion due to actin exposure following injury allows inflammatory mediators to cause widespread tissue damage [10].

Although tissue injury has not been clearly documented in early sepsis, low pGSN levels have been reported in sepsis patients [6], and a recent paper reported reduced pGSN levels in surgical sepsis patients [11]. From the available information, whether pGSN depletion results from surgery, sepsis or a combination thereof is unclear. However, animal models of sepsis reveal pGSN depletion within hours of septic challenge, and repletion of pGSN concentrations with recombinant pGSN reduces septic mortality. Moreover, circulating actin is detectable in the septic animals, and pGSN replacement converts it from an aggregated to a more soluble state [12]. We therefore undertook a pilot study to determine whether actin appears in the circulation of septic humans and if pGSN decreases correlate with outcomes in non-surgical sepsis patients.

## Methods

### Clinical sepsis database and plasma samples

Human plasma samples were selected from a prospectively enrolled cohort of patients admitted to adult intensive care units at the Massachusetts General Hospital (MGH), and all aspects of the study were approved by the Institutional Review Boards of MGH and the Harvard School of Public Health. Written informed consent was obtained from all participants or their appropriate surrogates. Details of the study have been previously described [13]. Patients were considered for inclusion in the cohort if they had any risk factor for acute respiratory distress syndrome (ARDS), including sepsis, septic shock, trauma, aspiration, or multiple blood product transfusions. Patients were excluded if they were immunosuppressed, were under 18 years of age, if a comfort care directive was in place, or if they had chronic lung disease. Platelet poor plasma samples were generated from blood collected in EDTA-containing tubes obtained from patients within 24 hours of admission and stored at −80°C. We only included patients admitted between 2001–2003 to minimized differences in clinical practices and then excluded those patients who had recently undergone surgical procedures (within one week of intensive care unit admission), or had concurrent diagnosis of ARDS. Twenty-one subjects with sepsis or septic shock, as defined by consensus criteria [14], with available plasma samples and clinical data, including Acute Physiology and Chronic Health Evaluation (APACHE) III scores, from this database were identified for the current study. pGSN and albumin levels were measured blindly as described below, and the results correlated with 28-day mortality data documented by the study database. Circulating actin was assayed by immunoblotting as described below. In addition, normal control plasma samples were obtained from seven volunteers (ages 22–40, four males and three females) from our laboratory.

### Gelsolin and Albumin Measurements

pGSN was measured in triplicate by its ability to stimulate actin nucleation as previously described [15]. Gelsolin quantification by the actin nucleation assay correlates well with levels obtained from Western blotting measurements [9]. The assay is highly specific, as evidenced by virtually zero activity in plasma of LPS treated gelsolin-null mice [16]. Actin or lipids complexing to pGSN do not affect pGSN's actin nucleation activity [17].

Albumin levels were measured colorimetrically using a commercial kit (Stanbio, Boerne, TX) according to the manufacturer's instruction.

### Western Blot Analysis

Each plasma sample was diluted 1∶5 and subjected to Western Blot analysis for actin. Each sample was heated at 85°C for 3 minutes in SDS-sample buffer (Boston Bioproducts, Worcester, MA) then analyzed by SDS-PAGE using 12% Tris-Glycine Gel (Invitrogen, Carlsbad, CA) and transferred to PVDF membranes (Millipore, Bedford, MA). After blocking the membrane overnight in 5% non-fat dry milk in Tris-buffered saline (TBS) with 0.05% Tween 20, primary antibodies were added and incubated at room temperature for 1 hr. To assay for plasma actin, a rabbit polyclonal anti-actin antibody (A2103, Sigma, St. Louis, MO) was used at a 1∶2000 dilution. Bound primary antibodies were probed with HRP-linked anti-rabbit IgG's (Cell Signaling, Beverly, MA) at 1∶2000 dilution. Chemiluminescence of HRP was developed with LumiGLO (Cell Signaling, Beverly, MA). Exposed and developed photofilm was scanned (Hewlett-Packard ScanJet, Palo Alto, CA).

### Statistics

Summary data are presented as mean±SD. Differences between groups were compared with the Fisher exact test for dichotomous variables and the Student *t* test for continuous variables with a normal distribution. Pearson correlations were used to assess associations between continuous variables. Logistic regression was performed to identify risk factors for mortality in univariate analyses. In order to facilitate direct comparisons, pGSN levels and APACHE III scores were grouped into quartiles prior to analysis. Variables with p less than 0.1 in univariate analysis were then used as independent variables in a stepwise logistic regression analysis, with a p less than 0.05 criterion for retention of variables in the final model. The multivariate procedure was validated by bootstrap bagging with 1,000 samples as has been previously described [18]. In the bagging procedure, repeated samples were generated with replacement from the original set of observations. For each sample, stepwise logistic regression was performed entering the predictors with p less than 0.1 at univariate analysis. Those factors identified as significant predictors in 50% or more of the analyses (median rule) were considered reliably statistically significant at p<0.05. Regression analyses were performed using SAS version 8.0 (SAS Institute, Cary, NC). Receiver operating curve characteristics were performed using Prism 4 (Graphpad Software, La Jolla, CA).

## Results

21 non-surgical patients with plasma samples available for this study from our sepsis database were included in this study. The mean±SD age was 66±18 years and 61.9% were men. Subjects were critically ill with a mean APACHE III score of 75±27. Overall, 6 deaths occurred within 28 days of admission yielding a mortality rate of 29%. Descriptive data by survivor status are displayed in Table 1. Non-survivors had higher APACHE III scores and were more likely to be men.

**Table 1:** Patient demographics and clinical information.

	28 Day Mortality		
	Survivor	Non-Survivor	P Value
**N**	15	6	
**Age**	66±17	67±14	0.93
**Gender (M∶F)**	7∶8	6∶0	0.046
**APACHE III**	68±25	93±24	0.045

### Circulating actin is detectable in septic patients

**Figure 1:**
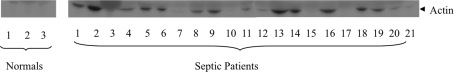
Actinemia occurs in human sepsis. Western Blot (WB) of plasma from septic patients and normals staining for actin shows that actin is present in the plasma of 17/21 patients, while Western Blot of three representative normals had no detectable actin in circulation.

Actin was identified in 81% (17/21) of plasma samples from septic patients but in none of 7 normal volunteers (p = 0.0003) (Figure 1). Circulating actin was detectable in 100% (6/6) of non-survivors but in only 73% (11/15) of survivors, although the difference was not statistically significant. In addition, pGSN levels and APACHE III scores did not differ significantly between patients with or without circulating actin. Because of species and isoform differences between the actins used as immunogens for the anti-actin antibodies and the blood samples examined as well as reference actin protein available for calibration, we could not precisely determine the actual amount of actin protein in the plasma samples. However, a minimal estimate based on comparing immunoblots of patient samples with purified human platelet actin is that actin-positive septic patient samples contained 25–50 µg actin protein/ml.

### Sepsis survivors had higher admission pGSN levels than non-survivors

**Figure 2:**
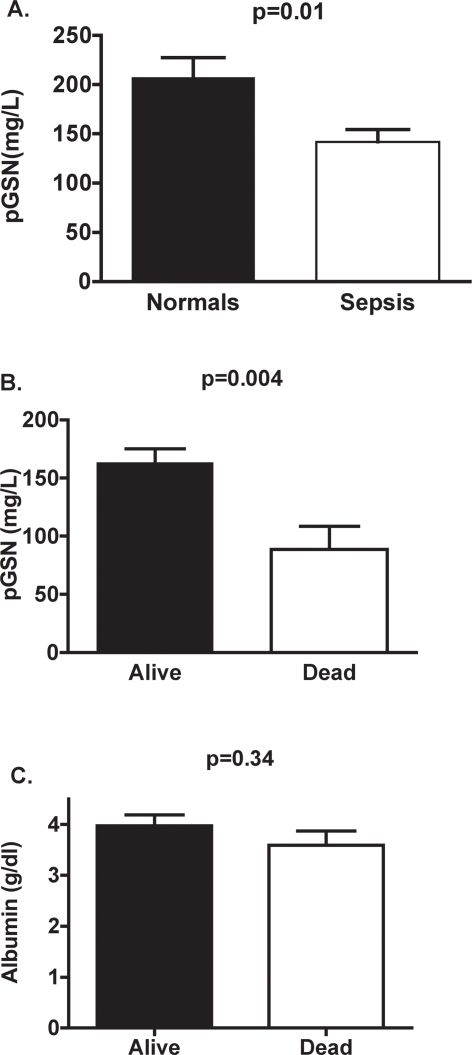
Degree of pGSN depletion is associated with sepsis mortality. A. Septic patients had lower pGSN levels compared to normal controls (p = 0.01). B. Septic patients who survived at 28 day of admission had significantly higher pGSN levels than those who did not survive (p = 0.004). C. Albumin levels did not differ between survivors and non-survivors.

The mean pGSN level in this cohort was 142 mg/L which is substantially lower than the average pGSN levels (207 mg/L) of our normal controls (p = 0.01, Figure 2A). The reported mean of pGSN is about 250 mg/L in a population of healthy controls [6]. Consistent with our hypothesis that pGSN levels reflect severity of illness, pGSN levels correlated inversely with Apache III scores, although the association did not achieve statistical significance (r = −0.35, p = 0.12). In addition, a trend was found for males to have lower pGSN levels than females (125±56 mg/L vs. 170±50 mg/L, p = 0.08), although this difference also did not meet criteria for statistical significance. No association was found between pGSN levels and either age or albumin levels.

pGSN levels were significantly lower in non-survivors compared to survivors (89±48 mg/L vs. 163±47 mg/L, p = 0.01) as shown in Figure 2B. The depletion of pGSN was not due to non-specific protein loss, since albumin levels were not significantly different between survivors and non-survivors (Figure 2C).

### Low pGSN levels predict mortality in sepsis patients

The OR for 28-day mortality was 3.44 (95% CI: [1.04–11.43]) for each quartile reduction in pGSN levels. In contrast, the OR for 28-day mortality with each quartile increase in APACHE III score was 3.28 ([0.97–11.1]), which was of borderline statistical significance (p = 0.06). In stepwise regression analysis including actin presence, APACHE III scores, albumin levels, age and pGSN levels, pGSN was the only independent mortality predictor.

**Figure 3:**
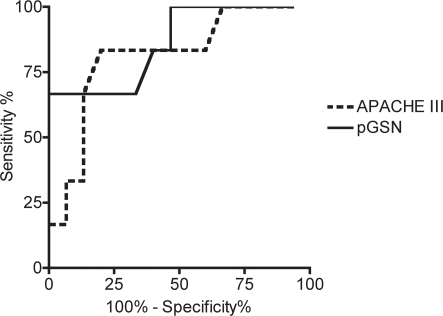
ROC analysis of pGSN and APACHE III. Receiver operating curves of pGSN levels (solid line) and APACHE III scores (dotted line) identifying 28-day mortality in septic patients are generated. Area under curve is 0.86 for pGSN (95% confidence interval of 0.68–1.05, p = 0.011), and 0.81 for APACHE III (95% confidence interval of 0.60–1.02, p = 0.03. A pGSN level cutoff of <113.6 mg/L yields a 66.7% sensitivity and 93.3% specificity with a likelihood ratio of 10.

Receiver operating curves (ROC) of pGSN levels and APACHE III scores with 28-day mortality as outcome is shown in Figure 3. pGSN levels showed moderate predictive ability with an area under the curve of 0.86 (p = 0.01). In contrast the area under the curve using APACHE III score to predict 28-day mortality was 0.81 (p = 0.03). Using a cutoff of 113.6 mg/L, pGSN has 66.7% sensitivity and 93.3% specificity for predicting 28-day mortality.

## Discussion

In this pilot study, we report for the first time that actin is detectable in plasma of non-surgical septic patients and that low pGSN levels strongly associate with mortality in these patients. We found that pGSN levels of non-surgical septic patients were significantly lower than our controls; however, it is important to note that our healthy controls were younger. The correlation between pGSN levels and outcome appears to be superior to APACHEIII, a severity score that incorporates a wide range of physiologic and clinical data. Our data suggest that pGSN depletion may be an important pathology of sepsis. This is consistent with the results of the study by Wang et al. examining surgical sepsis patients. Interestingly, Wang et al. reported much lower pGSN levels (0–50 mg/L in sepsis patients) compared to our study. Different methods of assaying pGSN (ELISA vs. functional nucleation) likely accounted for the difference; however, using the ELISA assay, Wang et al. reported much lower pGSN levels in healthy controls than those reported in the literature [2], [3], [6], [9], [19], [20]. It is possible that the ELISA method used by Wang et al. may underestimate true levels of circulating pGSN.

Based on its actin-binding property, pGSN has been categorized as part of the extracellular “actin-scavenging” system that counteracts actin toxicity when actin is released into the extracellular space [2]. Accordingly, the degree of pGSN depletion should reflect the degree of tissue injury that may lead to significant exposure of actin to the extracellular space. Indeed, low pGSN levels associate with poor outcomes in trauma [9] and critically ill surgical patients [8], [11], and in patients who received a cytotoxic conditioning regimen prior to hematopoietic stem cell transplantation [7].

Our observation that circulating actin is detectable early in septic patients suggests that tissue injury occurs at or near the onset of sepsis. Similar observations have been made in animal models of sepsis [12]. The origin of the circulating actin in sepsis is not clear; however, microparticles generated from circulating blood cells and endothelium in sepsis are possible candidates [21], [22]. Further investigation is warranted to test this speculation and to identify the location of the initial damage incurred in sepsis as the source of circulating actin.

In addition to protein molecules such as actin, A-β protein [23], and fibronectin [24], pGSN binds and modulates bioactive lipids, such as endotoxin [25], lysophosphatidic acid (LPA)[26] and platelet activating factor (PAF) [10]. pGSN can interfere with PAF's ability to activate platelets and neutrophils [10]. This effect may partially explain how exogenous pGSN replacement significantly enhances survival of septic animals [12], and blunts the inflammatory response in animal models of lung injury [27] and burns [28]. Based on these data, we propose that pGSN functions as an important endogenous guard against overwhelming inflammation from tissue injuries. Therefore, a pGSN deficient state associated with sepsis may be a modifiable risk factor for increased mortality and morbidity. Additional studies are needed to explore pGSN's importance in sepsis.
